# Protocol for a randomized controlled trial to evaluate the efficacy of inhibitory control training for aggressive behaviours among individuals with co-occurring substance use disorder and gambling behaviour

**DOI:** 10.1186/s13063-026-09503-y

**Published:** 2026-02-06

**Authors:** Yashita Ahluwalia, Siddharth Sarkar, Gauri Shanker Kaloiya, Vishal Deo, Swarndeep Singh, Swati Kedia Gupta, Yatan Pal Singh Balhara

**Affiliations:** 1 National Drug Dependence Treatment Centre (NDDTC), All India Institute of Medical Sciences, New Delhi, India; 2 Indian Council of Medical Research - National Institute for Research in Digital Health and Data Science, New Delhi, India; 3 Vardhman Mahavir Medical College and Safdarjung Hospital, New Delhi, India; 4 Department of Psychiatry, All India Institute of Medical Sciences, New Delhi, India; 5 Behavioral Addictions Clinic (BAC) and Centre for Advanced Research On Addictive Behaviors (CAR-AB), National Drug Dependence Treatment Centre and Department of Psychiatry, All India Institute of Medical Sciences, New Delhi, India

**Keywords:** Behavioral addictions, Gambling, Substance use disorders, Aggression, Inhibitory control training, Cognitive bias modification, Cognitive training

## Abstract

**Background:**

Aggression, substance use, and gambling behaviour often co-occur in a larger pattern of dysregulated behaviour. One of the factors that may underlie this phenomenon is impaired inhibitory control. Inhibitory Control Training (ICT) is an alternative intervention being tested for addressing addictive behaviours by targeting inhibitory control. Given the shared underlying mechanisms of these behaviours, applying ICT to aggression represents a possible extension of this intervention. This research will mark the first implementation of a cognitive bias modification approach to managing aggression among patients with co-occurring substance use disorder and gambling behaviour in the Indian context. A secondary aim of this study will be to assess whether changes in aggression and inhibitory control will be associated with reductions in substance use disorder and gambling behaviour.

**Methods:**

A two-group, parallel randomized controlled trial will be carried out in 130 male in-patients at a tertiary care centre. Participants fulfilling the DSM-5 criteria for substance use disorders, and screening positive for problem (Score ≥ 1) or pathological (Score ≥ 5) gambling on the South Oaks Gambling Screen will be randomly allocated to either experimental (ICT) or control (Sham Training) group. The ICT consists of six 15-min sessions over three days, using a Go/No-Go paradigm with 100% contingency. ST is matched with the active training in terms of both stimulus exposure and response requirements, but reduces the contingency agreement to 50%, thereby preventing the formation of associations between aggressive stimuli and inhibitory responses. Primary and secondary outcome measures will be assessed at baseline, 1-day, 1-month, and 3-months post-intervention.

**Discussion:**

As aggression is frequently observed in individuals with addictive behaviors and is closely linked to deficits in regulatory behaviour, the intervention, if proven efficacious, could offer a cost-effective and time-efficient alternative to traditional cognitive-behavioral and anger management interventions. Conversely, should the intervention prove ineffective, the findings would indicate that this may not be a potential area for further exploration in this population.

**Trial registration:**

The study protocol was registered prospectively with the Clinical Trials Registry of India (CTRI) on August 07, 2024 (Registration Number: CTRI/2024/08/072033) (URL: https://ctri.nic.in/Clinicaltrials/rmaindet.php?trialid=113292&EncHid=61291.44954&modid=1&compid=19).

**Supplementary Information:**

The online version contains supplementary material available at 10.1186/s13063-026-09503-y.

## Introduction

### Background and rationale {9a}

The dual process model of addiction explains it as a syndrome of dysregulated motivation, with a parallel interplay of dominating automatic, impulse-driven behaviours and deteriorating cognitive regulatory processes [1, 2]. In line with this theoretical proposition, research has targeted implicit mechanisms for reducing the extent to which individuals engage with their addictive tendencies. Impulse-driven behaviors have been targeted by attentional bias modification [3], approach-avoidance training [4], and interpretation bias training [5]. And the reverse has also been attempted with interventions targeting deficits in regulatory behaviors such as inhibitory control [18].

**Fig. 1:**
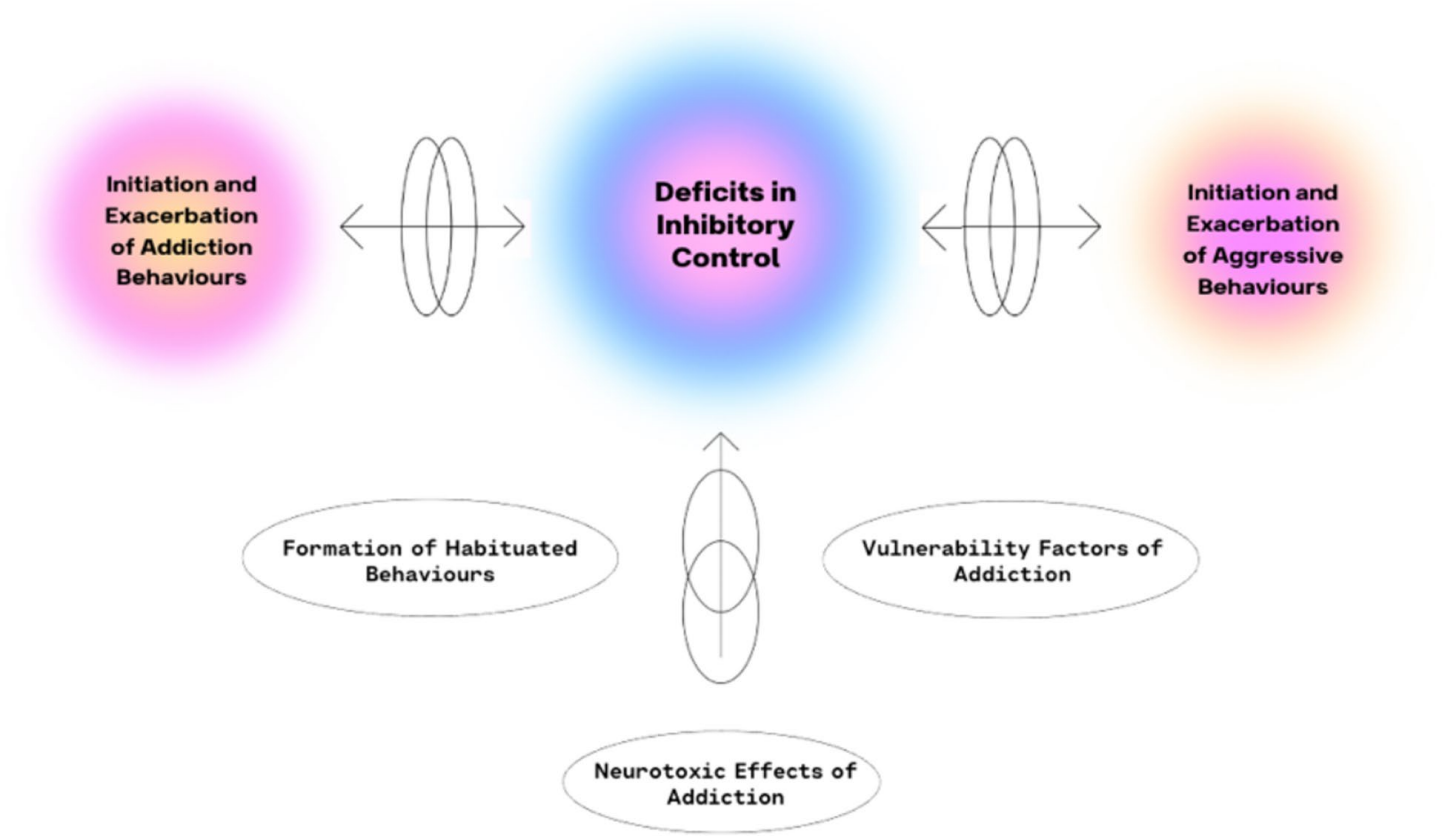
Interplay of inhibitory control, addiction, and aggressive behaviours

Empirical research has supported the notion that impairments in inhibitory control contribute to addictive behaviors and have been linked with both substance use disorders and gambling behaviour [6, 7]. However, this lack of inhibitory control may not stay confined to addiction. It may also extend to and impact other psychological domains that depend on the same regulatory mechanisms, such as aggression [8, 9]. Aggression is also frequently reported among individuals with substance use and gambling problems [10–12], lending further support to this link. Together, these findings point to a cycle where impaired inhibitory control, aggression, and addictive behaviors interact in a loop of cause and consequence (see Fig. 1).

This hypothesis is also in line with the problem behaviour theory [13] which proposes that gambling, substance use, and violent behaviours commonly co-occur in a ‘complex multi-condition behavioural syndrome’; creating a non-isolated, interrelated pattern of maladaptive behaviour. This has also been supported by subsequent empirical research [12–16].

The behaviour of individuals with low inhibitory control is often dominated by impulsive precursors such as unconscious patterns and automatic behaviour, which tend to bypass deliberate cognitive processing [17]. These individuals may react reflexively to internal urges and external stimuli especially under stressful conditions without adequately considering the consequences of their actions, leading to maladaptive responses. Such responses can vary depending on personal vulnerabilities and contextual factors. Common manifestations include aggression, continuing or relapsing into addictive behaviours, or engagement in other forms of risky decision-making [17].

In the field of addiction, this has led researchers to employ Inhibitory Control Training (ICT) to target these deficits through computerized behavioural interventions in an attempt to effectively reduce the frequency of substance consumption in participants [18–20]. Around 16 studies have explored this form of treatment all over the world by utilizing the go/no go paradigm or the stop signal paradigm in addressing substance use disorders. Building upon this approach, similar paradigms have been applied in the context of gambling behaviour, with Verbruggen et al. [21] and Verbruggen et al. [22] investigating the effects of proactive motor control training on gambling behavior. These approaches focus on strengthening inhibitory control via the association of no-go or stop cues and addiction-related stimuli. However, the results have been inconclusive and with mixed findings so far, possibly due to methodological heterogeneity across studies. Some of these studies have used sham training as a comparator where stimuli are presented in a way that prevents any form of association.

The application of ICT has not yet been extended to other psychological domains that are also influenced by deficits in inhibitory control. Since impaired inhibitory control can contribute to the inability to regulate aggression, which is strongly prevalent in people with substance use disorders and gambling behaviour; employing ICT could potentially address aggressive behaviors in this target population. Addressing this gap, the current research seeks to assess the effectiveness of ICT in decreasing aggressive behaviours and enhancing behavioural regulation among persons with substance use and gambling disorders. By extending the use of ICT beyond addiction-related outcomes, this research can contribute to understanding its utility as a transdiagnostic intervention, which can potentially be used in managing co-occurring maladaptive behaviours.

Aggression has been classically treated using cognitive-behavioral approaches and anger management training. Although effective, these treatments are usually time-consuming and require specialized training. In contrast, Inhibitory Control Training can be a time-efficient intervention, working on inhibitory control through underlying unconscious processes, to reduce aggressive tendencies. Such cognitive modification interventions have not yet been tested much among the Indian population. This research will mark the first implementation of ICT in an attempt to manage aggression among patients with co-occurring substance use disorder and gambling behaviour in the Indian setting.

### Explanation for the choice of comparator {9b}

ST will be matched with active training in terms of both stimulus exposure and response requirements, but will have the contingency agreement reduced to 50%, thereby preventing the formation of associations between aggressive stimuli and inhibitory responses. Similar studies in the past have used sham training as a comparator where stimuli were presented in a way that prevents any form of association [20, 23, 24]. Some other studies working with a different population have also shown the same [25]. Another reason for the inclusion of sham training in the control group is that while controlling for time and attention, it might provide information into the underlying mechanism of functioning of this intervention—differential effects between the groups, if observed, can be attributed to the formation of implicit inhibitory associations.

### Objectives {10}

The primary objective of this study will be to evaluate the efficacy of Inhibitory Control Training (ICT) in reducing overt aggressive behaviours among individuals with co-occurring substance use disorder and gambling behaviour. Additionally, the study aims to assess whether reductions in aggression and improvements in inhibitory control will be associated with subsequent changes in substance use patterns and gambling behaviour over follow-up periods.

## Methods: patient and public involvement, and trial design

### Patient and public involvement {11}

Patients and members of the public will not be directly involved in the reporting of this trial. However, the development and validation of the intervention employed in this randomized controlled trial will be informed by the target population. Patient feedback obtained during routine clinical interactions at the study site will be used to refine task instructions, session duration, and the overall feasibility of implementation. In addition, participant input will be incorporated during the pre-testing and cognitive interviewing stages to be undertaken as part of the translation of the study questionnaires.

### Trial design {12}

The research study will utilize a two-group, parallel, superiority randomized controlled trial (RCT) design. The primary objective of the trial is to determine whether inhibitory control training produces a greater reduction in aggressive behaviour compared to sham training, thereby testing the superiority of the experimental intervention over the comparator. Although the trial is designed as a superiority study, some aspects of it may be exploratory in nature due to our interest in whether the intervention works and how it works. Eligible participants will be randomly allocated in a 1:1 ratio to either the experimental group or the control group. In the experimental group, participants will receive inhibitory control training, while those in the control group will receive a sham version of the training. Both groups will also be receiving treatment as usual (TAU), as decided by the clinical team. Evaluation will occur at four time points: Pre-intervention (baselines), post-intervention at 1-Day post ICT, 1-month post ICT (± 4 Days), and 3-months post ICT (± 7 Days). The trial is designed to evaluate the superiority of Inhibitory Control Training over sham training in reducing aggressive behaviours.

## Methods: participants, interventions and outcomes

### Trial setting {13}

The participants will be recruited from the In-Patient Department (IPD) of the National Drug Dependence Treatment Centre (NDDTC), All India Institute of Medical Sciences (AIIMS), New Delhi.

### Characteristics of the people who are needed for the trial

**Table:** 

Characteristic	The people we would expect to see included
Age	Adult participants aged 18 years and above; age will be recorded in years and summarised using mean and standard deviation
Sex	Only male participants will be included in the study
Gender	No other gender identities will be included or reported in this study other than males
Race, Ethnicity and Ancestry	Not specifically collected or categorised; participants are recruited from the Indian population
Socioeconomic Status	Participants will not be included or excluded based on their socioeconomic backgrounds; however, socioeconomic details will be recorded using Modified Kuppuswamy Socioeconomic Scale (2023)
Geographic Location	Participants will be recruited from a tertiary care inpatient facility in Ghaziabad, Haryana, India. No eligibility criteria will be applied based on place of birth, duration of residence, or rural–urban background; however, these details will be recorded for descriptive purposes
Other Characteristics Relevant to the Trial	Adult male inpatients diagnosed with substance use disorder and exhibiting problem or pathological gambling behaviour; participants must be clinically stable, able to provide informed consent, and capable of engaging in brief computer-based behavioural training

### Eligibility criteria for participants {14a}

Male patients aged 18 years or older meeting the criteria for problem (Score ≥ 1) or pathological (Score ≥ 5) gambling on the South Oaks Gambling Screen (SOGS) [26], diagnosed with substance use disorder as per DSM-5 [27] criteria, and willing to give informed consent will be considered for inclusion in this study. Exclusion criteria include the presence of severe medical or psychiatric conditions that could interfere with participation, and a lifetime history of neurological illness, neurodevelopmental disorders, or traumatic head injury, as per the available clinical history.

The study has an inclusion criterion of male participants only because the inpatient population at the study site comprises a very small proportion of female patients. Including a highly unequal number of female participants would not allow for meaningful subgroup analyses and could introduce additional variability, thereby limiting the interpretability of the findings.

Participants will be recruited from an inpatient setting to ensure the feasibility of the intervention delivery. The training protocol requires two sessions per day over three consecutive days, due to which availability of participants across extended daytime hours will be necessary. Conducting the intervention in an outpatient setting might have led to logistical challenges. The inpatient setting was therefore decided upon to allow for feasible delivery of the intervention and minimized disruptions related to scheduling.

### Eligibility criteria for sites and those delivering interventions {14b}

The trial will be conducted at the inpatient facility of a tertiary care centre, which provides clinical services for the management of addictive disorders. The intervention will be delivered by the primary investigator (first author) of the study, who holds a Master’s degree in Clinical Psychology and will be undertaking this randomized controlled trial as part of her doctoral thesis.

### Who will take informed consent? {32a}

Written informed consent will be obtained from participants fulfilling the inclusion criterion by the primary investigator after providing a verbal explanation of the participant information sheet and addressing all questions. Participants who indicate their willingness to take part in the study will then be provided with the written consent form (see Additional file 4) for review and signature.

### Additional consent provisions for collection and use of participant data and biological specimens {32b}

Participants will be informed during the consent process that the study involves the collection and analysis of deidentified clinical and behavioural data only, and that no biological specimens will be collected. Consent will include permission for the use of deidentified data for the purposes of generating findings of this research study, and no other additional studies in the future. Participants will be informed that their data will be stored securely, accessed only by authorised members of the research team, and that they may withdraw consent for data use at any time without affecting their ongoing clinical care.

## Intervention and comparator

### Intervention and comparator description {15a}

The training program which will be administered to the experimental group will be an associative Go/No-Go Paradigm with 100% contingency. It is a computerized behavioral intervention that leverages implicit processes to associate no-go responses with aggression-related stimuli, thereby developing habitual links with the goal of reducing aggressive behaviors. The go cues (pictures contained in circles) will be consistently paired with non-aggressive stimuli, and the no-go cues (pictures contained in squares) with aggressive stimuli, maintaining 100% contingency. Participants will be instructed to respond to stimuli in accordance with the format of the pictures. They will be directed to respond to pictures contained in circles by ‘pressing the space bar’ and to withdraw from responding to pictures contained in squares. The presentation of stimuli will be interspersed with brief intervals of 1000 ms (ms) featuring cross slides, termed ‘inter-trial’ periods. The intervention will be presented on Microsoft® PowerPoint for Mac (Version 16.78.3) software. The training program will be structured into six sessions, spanning three consecutive days, with two sessions delivered on each day. These sessions will progressively increase in complexity [28], in terms of reducing the probability of ‘No-Go’ stimuli and decreasing stimulus response duration (see Table 1).

**Table 1:** Session-by-session plan for inhibitory control training

Training Session	Aggressive Stimuli (AS)		Non-Aggressive Stimuli (NAS)		Duration of Presentation (ms)	Total Time (Minutes)
	Proportion	Number [T (/C)]	Proportion	Number [T (/C)]		
Training Session—1	70%	35 (7)	30%	15 (3)	3000	15
Training Session—2	70%	35 (7)	30%	15 (3)	2500	15
Training Session—3	50%	25 (5)	50%	25 (5)	2500	15
Training Session—4	50%	25 (5)	50%	25 (5)	2250	15
Training Session—5	30%	15 (3)	70%	35 (7)	2250	15
Training Session—6	30%	15 (3)	70%	35 (7)	2000	15

*T (/C)* Total Pictures (Pictures Per Category), *ms* Milliseconds

Each session will consist of five blocks, and each block will comprise 50 trials. This structure will result in a total of 1500 trials throughout the training program. The choice of aggressive (no-go) and non-aggressive (go) stimuli employed in each training session will be determined by a Stimulus Evaluation Task, individualized to each participant [18].

**Fig. 2:**
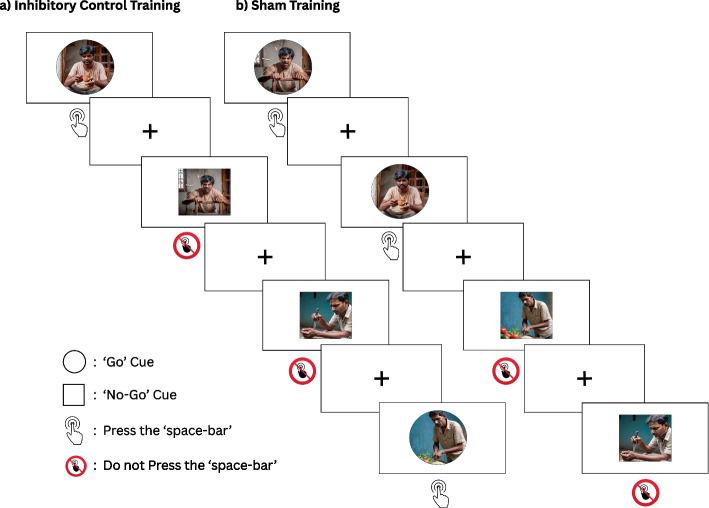
Panels represent the sequence of events during (**a**) Inhibitory Control Training and (**b**) Sham Training. The pictorial stimuli required for both training programmes shall be developed and validated in accordance with the Indian context for our target population

The sham training administered to the control group will employ an associative Go/No-Go Paradigm with 50% contingency instead of 100% (see Fig. 2). Go and no-go cues will be split equally between aggressive and non-aggressive stimuli. Participants will receive the same instructions and the same method of presentation of the intervention. During the practice sessions, participants will not receive feedback on correct and incorrect responses. These sessions will not progressively increase in complexity, as shown in Table 2. The training will also be organized into six sessions, spread out over three consecutive days, with two sessions per day. Each session will consist of five blocks, and each block will comprise 50 trials. This structure will result in a total of 1500 trials throughout the training program. The choice of aggressive (no-go) and non-aggressive (go) stimuli employed in each training session will be determined by a Stimulus Evaluation Task [18].

**Table 2:** Session-by-session plan for sham training

Training Session	Aggressive Stimuli (AS)		Non-Aggressive Stimuli (NAS)		Duration of Presentation (ms)	Total Time (Minutes)
	Proportion	Number [T (/C)]	Proportion	Number [T (/C)]		
Training Session—1	50%	25 (5)	50%	25 (5)	3000	15
Training Session—2	50%	25 (5)	50%	25 (5)	3000	15
Training Session—3	50%	25 (5)	50%	25 (5)	3000	15
Training Session—4	50%	25 (5)	50%	25 (5)	3000	15
Training Session—5	50%	25 (5)	50%	25 (5)	3000	15
Training Session—6	50%	25 (5)	50%	25 (5)	3000	15

*T (/C)* Total Pictures (Pictures Per Category), *ms* Milliseconds

### Criteria for discontinuing or modifying allocated intervention/comparator {15b}

The allocated intervention or comparator will be discontinued if a participant withdraws consent, and no data from such participants will be included in the analyses. If a participant leaves the facility against medical advice (LAMA) or is discharged by the treating clinical team before completing the intervention schedule, the intervention will be discontinued; however, all data collected up to that point will be retained, and participants will be contacted for follow-up assessments, subject to their willingness to continue participation. However, there are no special provisions for modification of the intervention or comparator once allocated because the intervention is non-invasive and low risk, and therefore the requirement for individual modifications based on participant response or tolerability is not anticipated.

### Strategies to improve adherence to intervention/comparator {15c}

Adherence to the intervention and comparator protocols will be facilitated by conducting the study in an inpatient setting, allowing for scheduled and in-person delivery of all sessions. Sessions will be administered at pre-specified times, and participants will receive verbal reminders prior to each session. Attendance and completion of sessions will be recorded by the intervention provider. All six intervention sessions will be delivered in person by the primary investigator, who will directly monitor adherence during the intervention phase. Following discharge, the primary investigator will contact participants and their designated family members using previously obtained contact details to remind them of upcoming follow-up assessments during the week preceding each scheduled follow-up.

### Concomitant care permitted or prohibited during the trial {15d}

All participants will continue to receive treatment as usual (TAU) for addictive disorder, as decided by the treatment team. TAU may include pharmacotherapy, brief psychosocial interventions, and supportive care. No aspects of standard clinical care will be withheld or restricted due to trial participation.

### Ancillary and post-trial care {34}

No specific ancillary or post-trial care beyond routine clinical management is planned as part of this study. Participants will continue to receive standard treatment services at the study site irrespective of trial participation or completion. As the intervention is non-invasive, low risk and exploratory in nature, no trial-specific compensation has been planned. In the event of any distress or adverse experience related to trial participation, appropriate clinical support and referrals will be provided by the treating team at no cost to the participant.

### Outcomes {16}

#### Study instruments for primary outcomes

The Overt Aggression Scale‐Modified [29, 30] is a semi-structured interview designed to evaluate various categories of aggressive behaviors. Four sub-components make up the overall aggression (AGG) score: Verbal Aggression, Aggression Against Object, Aggression Against Others, and Aggression Against Self. Subcomponent scores are determined by multiplying the number and severity level of aggressive behaviors of each type during the prior week. Additional scores include a Global Anger and Aggression (GAA) score composed of two item ratings: Subjective experience of anger and overt expression of anger. The OAS‐M has been shown to be reliable and valid, with good psychometric properties in terms of internal consistency, inter-rater reliability, test–retest reliability, face validity, convergent validity, divergent validity, and discriminant validity [30]. The primary outcome of the study will be the change in overall aggression score from baseline to the 1-Month post-intervention follow-up, as measured using the Overt Aggression Scale–Modified (OAS-M), and will be summarized using mean values and standard deviations.

#### Study instruments for secondary outcomes

The Inhibitory Control Task will be based on a cue-based ‘Frequent Go/Rare No-Go Paradigms’ [31]. Participants will be directed to respond to pictures contained in circles by ‘pressing the space bar’ and to withdraw responses to pictures contained in squares. The go cue will be presented with 100% consistency with non-aggressive stimuli, and the no-go cue with aggressive stimuli. The task will have 20% no-go cues and 80% go cues, in accordance with the frequent go/rare no-go arrangement, as presented in Table 3. The task will have a single session with five blocks, bringing it up to 250 trials. The task will be administered prior to and following the ICT. The task, prior to and post intervention, will be preceded by 10 trials of practice sessions utilizing non-aggressive stimuli in squares and circles as no-go and go cues, respectively. The participants will be provided feedback on incorrect and correct responses during the practice trials. The practice sessions will not be recorded or scored [18, 20, 32]. Commission and omission errors will be recorded. Based on the results of a research study estimating the reliability of associative Go/No-Go Tasks, our task with 50 trials per block is indicated to have an acceptable reliability between 0.60 and 0.80 [33].

**Table 3:** Details of one block of the inhibitory control task

Test	Aggressive Stimuli (AS)		Non-Aggressive Stimuli (NAS)		Duration of Presentation	Total Time
	Proportion	Number [T (/C)]	Proportion	Number [T (/C)]		
Pre-Test	20%	10 (2)	80%	40 (8)	1500 ms	15 Minutes
Post-Test	20%	10 (2)	80%	40 (8)	1500 ms	15 Minutes

*T (/C)* Total Pictures (Pictures Per Category)

The Stimulus Evaluation Task is an explicit, self-paced task which will instruct the participants to evaluate the aggressive pictures on an 11-pointer scale (0 to 10). The selection of pictures utilized during the training sessions and the main task will be determined by the patient’s rating on the Stimulus Evaluation Task. For the ICT, the highest-rated pictures will be used and for the sham training, the lowest-rated pictures will be employed. The 15 pictures not covered during the training will be included in the pre-test and post-test task to investigate the occurrence of generalization, defined as the transfer of inhibitory learning to untrained aggressive stimuli, which may indicate potential generalization of effects beyond the training context to real-world situations [18].

Information will be collected on the type and categories of substances consumed using a semi-structured performa for substance use details. Details regarding the routes of administration will be recorded, along with the age at which substance use began and the total duration of use. Data will be collected on the usual amount consumed per occasion and the typical frequency of use, reported in terms of times per day. The pattern of use will be assessed and characterized based on consistency and compulsivity. Additionally, the individual's involvement with support programs such as Alcoholics Anonymous will be documented, including the duration of participation. The date or time since the most recent substance use will also be noted during the assessment. The proforma was constructed by deriving questions from pre-existing literature focusing on similar areas (see Supplementary File 2).

The type and category of gambling behavior(s), along with the age of onset, duration, frequency, and pattern of engagement, will also be recorded using a semi-structured performa. It will also note the most recent episode of gambling. The financial information including the net amount spent, net losses, and net gains over time, will also be recorded. The proforma was constructed by deriving questions from pre-existing literature focusing on similar areas (see Supplementary File 2).

This South Oaks Gambling Screen (SOGS) [26] will be used to measure the type and the frequency of gambling. It consists of 20 multiple-choice questions that explore several aspects of gambling behavior, including consequences of the activity and its impact on interpersonal relationships and finances. The questionnaire covers a range of gambling activities, including playing cards, dice games, casino gambling, lotteries, bingo, stock market investments, commodities trading, slot machines, poker machines, skill-based games, pull tabs, and wagering on animals or sports events. The results of a validation study provide evidence of construct validity of the English version of the SOGS for screening gambling problems in a multiracial Asian community sample [34]. Even so, to maintain comprehensibility and consistency in questioning, the scale will be translated into Hindi following the World Health Organization (WHO) protocol incorporating the stages of forward translation, expert panel, backward translation, and pre-testing and cognitive interviewing.

The Timeline Followback (TLFB) [35] method is a retrospective assessment tool. It involves asking clients to retrospectively estimate their substance use seven days to two years prior to the interview date. Results showed high test–retest reliability and intraclass correlation coefficients for the scale [36], along with acceptable levels of convergent and discriminant [35].

The Alcohol, Smoking and Substance Involvement Screening Test (ASSIST V3.0) questionnaire has eight questions that rate use of different substances like tobacco, alcohol, cannabis, cocaine, amphetamine-type stimulants, inhalants, sedatives, hallucinogens, opioids, and 'other drugs'. It gives a risk score for each substance of use, ranging from 'low risk', 'moderate risk', to 'high risk' [37]. Studies demonstrate that ASSIST has good to excellent reliability, along with good concurrent, construct, predictive, and discriminant validity [38, 39].

Detailed information on each instrument’s specific measurement variable(s), analysis metric, method of aggregation, and time point for each outcome has been provided in Additional file 2 for primary outcome variables (Table S1) and secondary outcome variables (Table S2).

#### Harms {17}

No serious harms are anticipated from participation in this trial, as the intervention involves brief, computer-based behavioural training. Potential minor effects may include fatigue and frustration due to repetitive sessions or mild emotional discomfort due to aggressive stimuli displayed during the task. Still, any potential harms or unintended consequences arising during the trial, whether or not related to the intervention, will be assessed non-systematically through participant self-report and clinical observation during intervention sessions and assessments. Any adverse events reported or observed will be documented and communicated to the treating clinical team with no classification or reporting criterion, and the participant will be free to discontinue engagement with the study whenever they may want to. Any harms identified during the trial will be reported descriptively in trial publication.

#### Participant timeline {18}

Patients admitted to the National Drug Dependence Treatment Centre (NDDTC) will be administered upon a brief screening process. The screening will initiate only when the participant is able to actively engage in the study, ensuring they are not intoxicated or in a state of withdrawal. Withdrawal charting will be done using the Clinical Institute Withdrawal Assessment of Alcohol Scale, Revised (CIWA – Ar) [40] for alcohol, Clinical Opiate Withdrawal Scale (COWS) [41] for opioids, Minnesota Nicotine Withdrawal Scale (MNWS) [42], the Cannabis Withdrawal Scale (CWS) [43] for cannabis, and the Clinical Institute Withdrawal Assessment Benzodiazepines (CIWA—B) [44] scale for Benzodiazepines. If the participant is found intoxicated or experiencing withdrawal, the screening process will be paused. It will be resumed only after a reassessment of withdrawal symptoms has been conducted within the next 24–48 h.

Patients diagnosed with substance use disorder by a psychiatrist will then undergo the South Oaks Gambling Screen (SOGS) for the identification of problem or pathological gambling in the patients. Once it is ascertained that the participant fulfills the inclusion/exclusion criteria, written consent will be taken by the primary investigator, and patients will be randomized to either the experimental or control group. The Alcohol, Smoking, and Substance Involvement Screening Test (WHO-ASSIST Version 3.0) and the Overt Aggression Scale – Modified (OAS – M) will be administered along with the sociodemographic and clinical proformas. Other baseline assessments, including the Stimulus Evaluation Task, Inhibitory Control Task, and the Timeline Follow-Back Scale (TLFB) will also be performed post randomization. All questionnaires and assessments will be administered by verbally posing the questions (and options) to the patients. The demographic details, medical/psychiatric history of the participant, their family history, and treatment record will also be recorded (see Supplementary File 2).

Subsequently, the experimental and control group participants will undergo the inhibitory control training or sham training for 3 consecutive days, respectively, administered by the primary investigator (psychologist) for all patients. Throughout this period, the study participants will continue to receive concurrent treatment as usual as per the advice of the clinical treating team. Any changes in the medication that occur during the study period will be recorded.

**Fig. 3:**
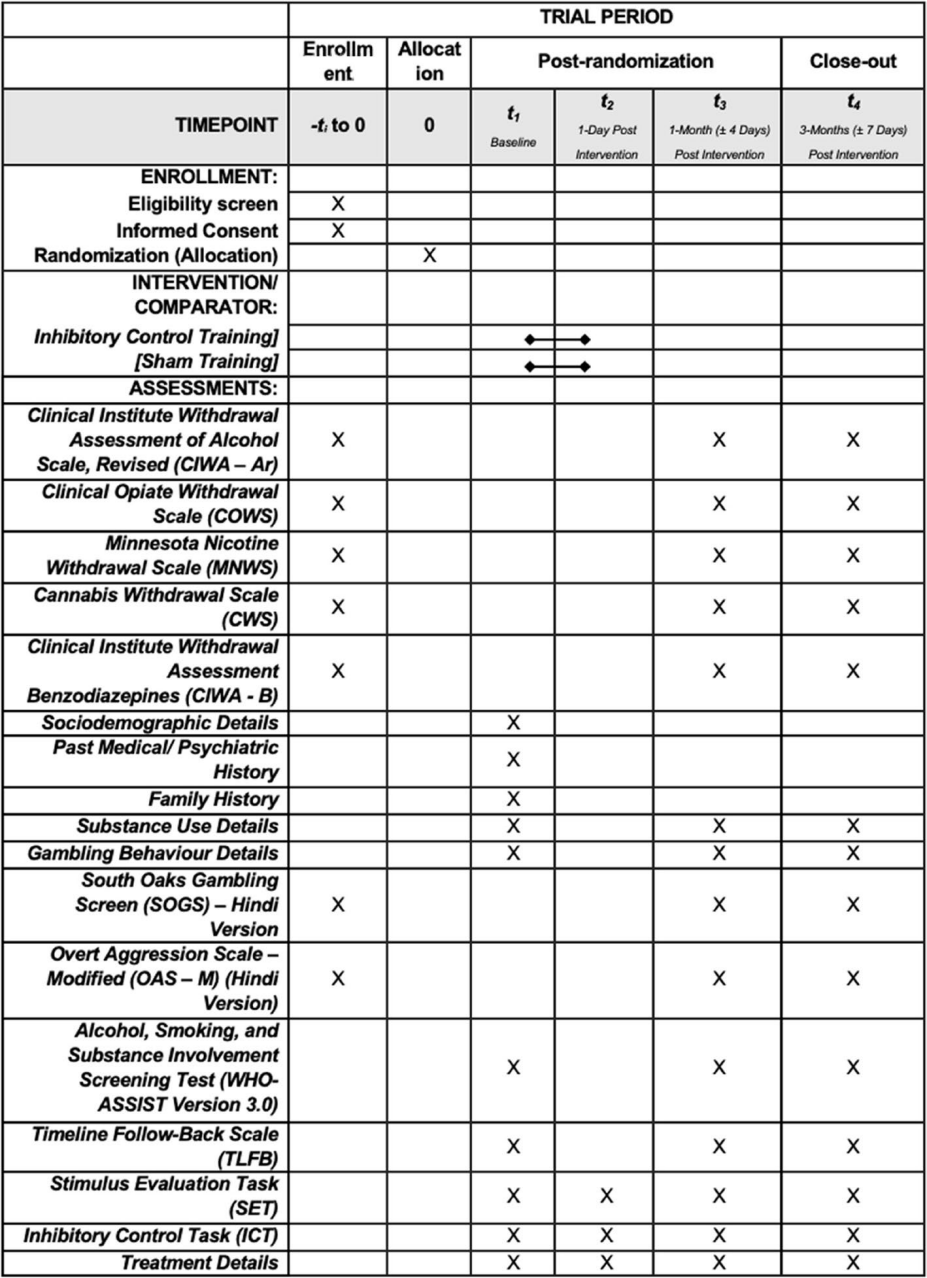
Participant timeline: schedule of enrollment, interventions, and assessments

One day after the intervention, participants will be assessed using the outcome measure administered during the baseline assessment. The first follow-up will be held at 1 month post-intervention (± 4 Days), and the second follow-up will be held at 3 months post-intervention (± 7). A comprehensive record of treatment prescribed to the participants will be maintained throughout the study period. After the final follow-up, participants will be prompted to guess their allocation group, either control or experimental, which will aid in assessing the effectiveness of the trial's blinding. They will also be asked about their perception of whether the intervention they received influenced their levels of aggression. The instrument administration and study timeline has been summarized in Fig. 3.

Written informed consent will be obtained from all the participants prior to enrolment by the primary investigator. No invasive procedure(s) will be conducted on the participants. All the information gathered in the course of the study will be kept confidential. Participation or non-participation in the study would not affect the usual treatment of the participants. The participants can withdraw their consent for participation in the study at any point. Participants will be informed that they will undergo either an active or sham training over three days, with assessments before, after, and during follow-ups at 1 and 3 months. Participation is voluntary, with no assured benefits or risks anticipated, and all data will be kept strictly confidential. While the intervention is intended to help, there is a possibility it may not produce the desired therapeutic effect. They will be informed that the sham training is designed to resemble the actual intervention without offering any therapeutic benefit.

During the course of study participation, any increase in distress or complaints noted or reported by the participant will be communicated to the treating team, and necessary referrals will be made. Any such adverse events occurring during the study period will be recorded and reported as per standard reporting guidelines.

#### Sample size {19}

Drawing upon findings from Khan et al. [45], patients exhibited a significant reduction in aggression from a mean of 3.44 (SD = 3.89) to 1.07 (SD = 1.92) on the Overt Aggression Scale—Modified (OAS-M) after the computerized cognitive and social cognition training. The effect size was calculated using the G*Power software (Version 3.1.9.6) with a priori power analysis for a t-test comparing two dependent means (matched pairs) and an assumed correlation of 0.5 between paired measurements [46]. A paired t-test framework was used for effect size estimation because the control group (sham training) in this study serves primarily as a means of studying the mechanism of work of the ICT (i.e., 100% contingency-based associative learning), due to which we employed only 50% contingency between aggressive stimuli and no-go responses in the ST, which as suggested by some of the past literature, opposes how the ICT works [20, 23, 24]. However, recent studies have reported comparable effectiveness of sham and active inhibitory control training, raising uncertainty regarding the specificity of the proposed mechanism [47]. Similar concerns have also been raised in relation to attentional bias modification interventions, which were previously assumed to operate through similar mechanisms but have more recently been shown to give non-specific effects [48]. Therefore, to estimate the expected magnitude of change attributable to inhibitory control training itself, pre–post scores from the experimental condition were used for effect size calculation from prior literature.

According to the above estimates based on the prior literature, with a corresponding effect size of 0.70, power of 80%, and alpha of 0.05, the necessary sample size required to detect a comes out to be at least 26 subjects in each group (i.e., experimental and control) [49].

As per another study conducted at the same tertiary care centre as the current study [50], the dropout rate among patients was about 45% at 1 month, and another 15% increase in dropouts was reported at the 3-month follow-up. Thus, assuming a planned attrition rate of 60% in both groups using this reference, the overall required sample size for the research study is recalculated to 130 participants, with 65 participants in each group.

#### Recruitment {20}

Regular screening for inclusion and exclusion criteria will be conducted in the inpatient department of the recruitment site several times per week to identify newly admitted patients who meet the eligibility requirements.

## Assignment of interventions: randomisation

### Sequence generation: who will generate the sequence {21a}

The random allocation sequence will be generated by an independent biostatistician who is not involved in participant recruitment, intervention delivery, or outcome assessment. The allocation sequence will be generated using the R software [51].

### Sequence generation: type of randomisation {21b}

Restricted randomisation using permuted block randomisation will be employed to ensure balanced allocation between the experimental and control groups. Randomized block sizes of 4, 6, and 8 will be used to reduce predictability of the allocation sequence. No stratification factors will be applied.

### Allocation concealment mechanism {22}

Allocation concealment will be ensured using the Sequentially Numbered, Opaque, Sealed Envelopes (SNOSE) [52] method. The random allocation sequence will be placed into sealed envelopes by a researcher not involved in participant enrolment or intervention delivery. Envelopes will be opened sequentially only after a participant has provided written informed consent.

### Implementation {23}

The primary investigator responsible for participant enrolment, intervention delivery, and outcome assessment will not have access to the random allocation sequence prior to participant assignment. Group allocation will be revealed to the primary investigator only at the time of opening the sealed envelope.

## Assignment of interventions: blinding

### Who will be blinded {24a}

Only trial participants will remain blinded to allocation throughout the study period, making it a single-blind study. Investigator blinding was not feasible, as all aspects of participant enrolment, intervention delivery, outcome assessment, and analysis are conducted by a single investigator.

### How will be blinding be achieved {24b}

Participant blinding will be achieved by ensuring that the experimental and sham training procedures are the same in appearance, duration, session structure, instructions, and mode of delivery. Both conditions will have identical computerized Go/No-Go tasks administered over six sessions across three consecutive days, using similar stimuli and response requirements, but different association contingencies. Participants will be informed that they will receive either an active or a sham version of the training, without disclosure of the specific feature of contingency distinguishing the two conditions.

Given that the intervention is designed to operate through implicit learning mechanisms by unconsciously forming associations between aggressive stimuli and inhibitory responses, it would not be appropriate to explicitly inform participants that the intervention targets aggression. Hence, the participant information sheet has described the study as follows: *“You are invited to participate voluntarily in a research study focused on providing a psychological intervention aimed at alleviating symptoms associated with substance use through the use of visual stimuli.” This will be cleared out at the end of the study when the participants will be informed regarding the true nature of the study and why it was required by the investigators to avoid disclosure of the same, at the beginning.* Prior to this and following the final follow-up, participants will be asked to guess their allocation group, either control or experimental, which will aid in assessing the effectiveness of the trial's blinding. They will also be asked about their perception of whether the intervention they received influenced their levels of aggression.

### Procedure for unblinding if needed {24c}

Unblinding of participants is not anticipated during the course of the trial, as the intervention is non-invasive and low risk. However, if carried out, any instance of unblinding will be documented, along with the reason and timing of disclosure. The primary investigator will have access to the group allocation of all enrolled participants.

## Data collection and management

### Plans for assessment and collection of outcomes {25a}

The outcome measures will be assessed at four time points: baseline (pre-intervention), 1-day post-intervention, 1-month post-intervention, and 3-months post-intervention. Pre-specified assessment windows have also been defined as follows: post-intervention (1 day), 1-month follow-up (± 4 days), and 3-month follow-up (± 7 days). All assessments will be administered by the primary investigator using standardized instruments, including the Overt Aggression Scale – Modified (OAS-M), Inhibitory Control Task, South Oaks Gambling Screen (SOGS), Alcohol, Smoking and Substance Involvement Screening Test (ASSIST v3.0), Timeline Follow-Back (TLFB), and the Stimulus Evaluation Task. These instruments have acceptable reliability and validity, with details of their psychometric properties described in the Methods section. To ensure consistency in questioning, questionnaires will be administered in Hindi following standardized translation procedures. All questionnaires will be asked in-person using an interview-style format with all participants to ensure that the data is complete and accurate. Data collection forms can be made available upon reasonable request to the corresponding author via email (ypsbalhara@gmail.com).

The semi-structured proformas to be used in this study have been added to Additional file 3. The questionnaires for the Stimulus Evaluation Task, the Inhibitory Control Task, and the Inhibitory Control Training will be specifically developed for use in this study. Other questionnaires developed by other researchers to be employed in this study will also be appended to the same file—These questionnaires were either free to use or permission to use them in our study has been obtained from their authors.

Separate data collection forms will be created for baseline and for each follow-up time point. All data collection forms will be prepared using Microsoft® Word for Mac (Version 16.104) and stored in individual Word files for every participant. All assessments will be conducted in person by the primary investigator using an interview format. Questionnaires will be checked for completeness at the time of administration, allowing any missing or unclear responses to be clarified immediately.

### Plans to promote participant retention and complete follow-up {25b}

Participant retention will be promoted by conducting the intervention phase in an inpatient setting, allowing supervised completion of all training sessions. Follow-up assessments will be scheduled in advance, and participants and their designated family members will be contacted using previously obtained contact details to provide reminders during the week preceding each follow-up. Follow-up time points have been set up with windows to accommodate participants’ availability.

## Data management {26}

Data from our electronic word files will be copied and stored in Microsoft® Excel for Mac (Version 16.104) by the primary investigator. Periodic checks will be conducted for completeness and consistency. Range checks will also be implemented following data entry to ensure that the data values are appropriate. Final datasets will be secured and retained in accordance with institutional data management policies. All trial datasets will remain shared with the supervisor of the doctoral study (Corresponding Author).

## Confidentiality {33}

Personal and clinical information will be collected directly from participants during in-person assessments and recorded anonymously using unique study identification codes. Identifiable information, including consent forms and contact details, will be stored separately from study data in locked cabinets and password-protected digital devices accessible only to authorised members of the research team. Confidentiality will be maintained throughout the study and after its completion in accordance with institutional ethics guidelines.

## Statistical methods

### Statistical methods for primary and secondary outcomes {27a}

Initial analyses will summarize demographic and clinical data of the participants using descriptive statistics. Mean values and standard deviations will be used to describe baseline and outcome variables for the experimental as well as for the control condition across all assessment times. Prior to further analyses, t-tests (or equivalent non-parametric tests) will be applied to confirm equivalence between both groups in demographic characteristics and pre-intervention scores. Any initial differences will be considered for adjustment in subsequent analyses. Based on the normality of the data, parametric or equivalent non-parametric tests will be conducted to assess the differential effects of the intervention training compared to the sham training on each outcome variable over time. The primary outcome, overt aggression measured using the Overt Aggression Scale – Modified (OAS-M), will be analysed with time (baseline, 1-day post-intervention, 1-month post-intervention, and 3-month post-intervention) as the within-subjects factor and group (inhibitory control training vs sham training) as the between-subjects factor.

Secondary continuous outcomes will also be analysed using similar models. Outcomes expressed as categorical or binary variables (e.g., substance use or gambling presence) will be summarised as frequencies and proportions and analysed using appropriate analysis methods. All statistical analysis methds wil onl;y be decided upon once its ensures that our collected data meets the assumptions of the respective test. Harms will be reported descriptively.

### Who will be included in each analysis {27b}

The primary analyses will follow the intention-to-treat (ITT) principle, including all randomized participants in the groups to which they were originally allocated, irrespective of intervention completion or follow-up attendance. In addition, per-protocol analyses will be conducted as a secondary analyses, including only participants who complete all six intervention sessions and all follow-up sessions.

### How missing data will be handled in the analysis {27c}

The method of data imputation will be chosen based on the characteristics and nature of the missing data following completion of data collection, ensuring appropriate handling of any incomplete data points.

### Methods for additional analyses (e.g. subgroup analyses) {27d}

After the completion of data collection, the data will be reviewed for suitability of the following analyses: Post hoc comparisons may be conducted for detailed analysis through between group and within group measures in case prior statistical analysis indicates significant differences. The moderating effect of changes in inhibitory control and stimulus devaluation immediately post intervention on changes in overt aggression behaviours at 1-month and 3-months post-intervention may be analysed.

The moderating effect of changes in inhibitory control and aggressive behaviour immediately following the intervention and at 1-month post-intervention on changes in substance use frequency and gambling behaviour at 1-month and 3-months post-intervention may be analysed.

If a significant dropout rate occurs during the study, an analysis can be conducted to identify any patterns or trends in the characteristics of participants who dropped out. This analysis can help understand potential reasons for dropout in terms of demographic, clinical, or other factors associated with higher dropout rates.

### Interim analyses {28b}

No interim analyses are planned for this trial. Given the short duration of the intervention, the low-risk nature of the behavioural training, and the modest sample size, no formal stopping guidelines have been established. All analyses will be conducted after completion of data collection.

### Protocol and statistical analysis plan {5}

The full trial protocol, including the statistical analysis plan, is being submitted for publication and will be publicly accessible upon publication. The statistical analysis plan is integrated within the protocol document. The same data will also be available the Clinical Trials Registry of India (CTRI). Any substantive deviations from the pre-specified analysis plan will be clearly documented and justified in subsequent publications.

## Oversight and monitoring

### Composition of the coordinating centre and trial steering committee {3d}

This research study is being conducted as part of the doctoral thesis of the primary investigator. Oversight of the trial will be provided by a Doctoral Committee comprising seven members with expertise in psychiatry, clinical psychology, and/or biostatistics. This committee will be responsible for supervising the overall conduct of the trial, providing academic, methodological, and implementational guidance, and reviewing trial progress at scheduled meetings. No relevant decisions can be made with regards to the trial without consent from all 7 members of this committee. The protocol has also been formulated under the supervision of the doctoral committee.

### Composition of the data monitoring committee, its role and reporting structure {28a}

For this study, a separate Data Monitoring Committee has not been composed. Continuous monitoring of trial conduct and participant safety will be taken up by the Doctoral Committee supervising the doctoral thesis. The committee will review the progress of the study, discuss any concerns regarding data quality or participant safety, and advise the principal investigator.

### Frequency and plans for auditing trial conduct {29}

The Doctoral Committee shall convene a minimum of every six months to review the progress of trials, assess compliance with the approved protocol, and discuss operational or methodological problems identified during the study conduct. Issues noted during the review shall be documented and dealt with in a timely manner.

### Protocol amendments {31}

Any major protocol amendments, including changes to study design, eligibility criteria, interventions, outcomes, or analysis plans among others, will be submitted for approval to the Institutional Ethics Committee prior to implementation. Prior to this, proposed amendments will be reviewed and discussed during Doctoral Committee meetings. Relevant updates will also be communicated to the trial registry and documented in trial reports. They will also be communicated to the participants in case the revision(s) impact treatment and require the investigators to obtain an updated informed consent. Any deviations from the protocol will also be documented using a breach report form.

### Dissemination policy {8}

The results of the trial will be disseminated through publication in peer-reviewed scientific journals and presentation at relevant academic conferences.

## Discussion

This study will be the first attempt to examine the efficacy of Inhibitory Control Training (ICT) in reducing aggression among individuals with co-occurring substance use and gambling disorders in the Indian context. Drawing from the Problem Behavior Theory and the Dual Process Model of Addiction, the proposed intervention will target implicit mechanisms of behavior regulation by associating no-go cues with aggression-related stimuli. Unlike conventional therapies focusing on conscious reflection and decision-making, this paradigm makes use of unconscious processing to promote behavioral inhibition. As aggression is frequently observed in individuals with addictive behaviors and is closely linked to control and regulation, the intervention, if proven efficacious, could offer a cost-effective and time-efficient alternative to traditional cognitive-behavioral and anger management interventions. Conversely, should the intervention prove ineffective, the findings would indicate that this may not be a productive area for further exploration. Importantly, the study extends existing literature by contextualizing the intervention in India—a country where research on aggression and gambling in addiction populations remains underrepresented.

### Expected limitations and strengths

Among the strengths of the study is the use of a randomized controlled trial design that incorporates permuted block randomization and allocation concealment through the SNOSE method with well-structured active and placebo interventions. Another strength is the application of sham training on the control group which while controlling for time and attention, might also provide information into the underlying mechanism of functioning of this intervention. The use of multiple validated outcome measures ensures that all behavioural dimensions will be accurately recorded. Questionnaires are also being translated into Hindi, to maintain the consistency of questioning. Furthermore, assessments across four time points, including follow-ups at one-day post intervention, allow the investigation of both immediate and sustained effects. The individualized stimulus selection based on participant ratings may increase personal relevance, potentially increasing its effectiveness. Furthermore, the toolbox and the intervention prepared for this study can also be validated and applied on different populations, and for different treatment interventions.

However, along with the strengths, the limitations of the study must also be acknowledged. The study sample will be restricted to male inpatients with a specific diagnosis, which might limit its applicability to other populations. The same is the case with the development and validation of the study intervention. The intervention itself is brief, lasting only 3 days, and based on inconclusive mechanisms of work, which raises questions about its capacity in being capable of dealing with complex, chronic patterns of behavior. Lastly, the computerized nature of the task, although practical, may not be completely representative of real-world interactions of aggressive behavior.

### Expected outcomes

Despite these constraints, the research will inform us on the efficacy of this line of cognition-based interventional approaches in the Indian population. These kinds of interventions have not been tested in India before, and it might be useful to determine whether additional research along this line should be conducted. It is expected that individuals in the ICT group will exhibit significantly reduced overt aggression, as measured by the OAS-M, particularly at the one-month and three-month follow-ups. Improvements in inhibitory control are also anticipated. If proven efficacious, the underlying mechanism of the same may also be analyzed in terms of changes in the capacity for inhibitory control and ratings on the stimulus evaluation task. Given the interconnectedness of aggression, substance use, and gambling behavior, it will be assessed if reductions in aggression and improvements in inhibitory control may contribute to subsequent reductions in substance use frequency and gambling severity. Furthermore, participants in the ICT group may demonstrate generalization of the training effect to novel stimuli. This will be tested using ratings on other stimuli, and ratings on other stimuli not incorporated in the intervention.

In conclusion, this study has the potential to advance both theoretical understanding and clinical practice by introducing a resource-efficient intervention for a highly comorbid and underserved population. If successful, ICT may serve as an adjunct to conventional treatments, opening broader applications in addiction and behavioural health care.

### Trial status

The study protocol was developed as part of a three-to-five-year doctoral program starting from October 2023. The preparatory phase included constitution of a doctoral committee, development of the research protocol and subsequent modifications, followed by approval from the Institutional Ethics Committee and registration with the Clinical Trials Registry of India (CTRI). Participant enrolment was initiated on January 14, 2025, and is anticipated to complete in December 2026. Following this, analysis of the results and publication of the findings will be worked upon.

## Supplementary Information

Additional file 1. SPIRIT Guidelines Checklist.

Additional file 2. Details of the Primary and Secondary Outcome Measures.

Additional file 3. Semi-structured questionnaires constructed for the study.

Additional file 4. Informed consent documents given to the participants.

## Data Availability

The data can be made available upon completion and publication of the research study to researchers who provide a methodologically sound proposal to achieve the goals of the approved proposal. Any data required to support the protocol can also be supplied on request. Proposals should be submitted to the corresponding author.

## Abbreviations

ASSIST: Alcohol, Smoking and Substance Involvement Screening Test
CIWA-Ar: Clinical Institute Withdrawal Assessment of Alcohol Scale, Revised
CIWA-B: Clinical Institute Withdrawal Assessment for Benzodiazepines
COWS: Clinical Opiate Withdrawal Scale
CTRI: Clinical Trials Registry of India
CWS: Cannabis Withdrawal Scale
DSM-5: Diagnostic and Statistical Manual of Mental Disorders, Fifth Edition
ICT: Inhibitory Control Training
IPD: Inpatient Department
ITT: Intention-to-Treat
LAMA: Leave Against Medical Advice
MNWS: Minnesota Nicotine Withdrawal Scale
OAS-M: Overt Aggression Scale–Modified
RCT: Randomized Controlled Trial
SET: Stimulus Evaluation Task
SNOSE: Sequentially Numbered, Opaque, Sealed Envelopes
SOGS: South Oaks Gambling Screen
SPIRIT: Standard Protocol Items: Recommendations for Interventional Trials
ST: Sham Training
TAU: Treatment as Usual
TLFB: Timeline Follow-Back
