# Supplementary material for: Protocol for a randomized controlled trial to evaluate the efficacy of inhibitory control training for aggressive behaviours among individuals with co-occurring substance use disorder and gambling behaviour
Source: Trials. 2026 Feb 6;27:199. doi: 10.1186/s13063-026-09503-y (PMC12973630; doi:10.1186/s13063-026-09503-y)
Supplement: Supplementary file 1 — Additional file 1. SPIRIT Guidelines Checklist. [file 13063_2026_9503_MOESM1_ESM.docx]

**Table S1:** Details of the Outcome Measures and the Assessment Tools used to Assess the Primary Measures

| **Outcome Measure** | **Domains** | **Assessment Tool** |
| --- | --- | --- |
| **Inhibitory Control** | **Commission Errors**  Omission Errors | Inhibitory Control Task |
| **Aggression Levels** | Verbal Aggression Score  Aggression Against Objects Score  Aggression Against Others Score  Aggression Against Self Score  Total Aggression Score (Verbal, Object, Other, Self)  Global Subjective Anger  Global Overt Aggression | Overt Aggression Scale – Modified (OAS-M)^[27,28]^ -Hindi Version |
| **Perceived Aggressiveness** | Subjective aggressiveness rating (0–10 scale) for 50 aggressive images.  **Generalization Score** | Stimulus Evaluation Task |

**Table S2:** Details of the Outcome Measures and the Assessment Tools used to Assess the Secondary Measures

| **Outcome Measures** | **Sub-Categories/ Domains** | **Assessment Tools** |
| --- | --- | --- |
| **Gambling Behaviour Details** | Name(s) of Gambling Behaviour  Category of Gambling Behaviour  Age of Onset of Gambling Behaviour  Duration of Gambling Behaviour  Usual Frequency of Gambling Behaviour  Pattern of Gambling Behaviour  Total Number of Abstinent Attempts  Duration of Abstinent Attempts  Last Episode of Gambling Behaviour  Net Amount Spent in Gambling Behaviour  Net Loss in Gambling Behaviour  Net Profit in Gambling Behaviour | Semi-Structured Performa |
|  | Type of Gambling Activity  Mode of Gambling Activity  Frequency of Engagement  Social and Financial Consequences  Impact on Interpersonal Relationships - Attempts to Reduce or Stop Engagement  Insight into Gambling Activity | South Oaks Gambling Screen (SOGS)^[31]^ – Hindi Version |
| **Substance Use Details** | Name(s) of Substance  Category of Substance  Age of Onset of Use  Duration of Use  Usual Amount of Use  Usual Frequency of Use  Pattern of Use  Number and Duration of Abstinent Attempts  Last Intake | Semi-Structured Performa |
|  | Retrospective recall of quantity and frequency of Substance Intake | Timeline Follow-Back Scale (TLFB)^[32]^ |
|  | Use of 10 Substance Categories (Tobacco, Alcohol, Cannabis, Cocaine, Amphetamines, Inhalants, Sedatives, Hallucinogens, Opioids, Others) - Lifetime and Recent Use | Alcohol, Smoking and Substance Involvement Screening Test (ASSIST V3.0) |
