# Supplementary material for: Protocol for a randomized controlled trial to evaluate the efficacy of inhibitory control training for aggressive behaviours among individuals with co-occurring substance use disorder and gambling behaviour
Source: Trials. 2026 Feb 6;27:199. doi: 10.1186/s13063-026-09503-y (PMC12973630; doi:10.1186/s13063-026-09503-y)
Supplement: Supplementary file 2 — Additional file 2. Details of the Primary and Secondary Outcome Measures. [file 13063_2026_9503_MOESM2_ESM.zip › Additional File 2 R2.docx]

**Socio-Demographic Details**

**Patient Code: Training:**

| **Unique Health Identification** |  |
| --- | --- |
| **Name** |  |
| **Bed Number** |  |
| **Age** |  |
| **Date of Assessment** |  |
| **Date of Admission** |  |
| **Contact Number (Patient)** |  |
| **Contact Number (Informant)** |  |

| **Gender** | 1.Male | 2.Female | | 3.Other | | | |
| --- | --- | --- | --- | --- | --- | --- | --- |
| **Educational Qualification** | 1. Illiterate | 2.Less than Graduate | | 3.Graduate | | 4.Postgraduate | |
|  | Number of Years of Education: | | | | | | |
| **Marital Status** | 1. Never Married | 2.Currently Married | | 3.Seperated | 4.Divorced | | 5.Widow(er) |
| **Employment Status** | 1.Never Employed | 2.Presently Unemployed | 3.Full-Time Employed | 4.Part-Time Employed | 5.Self-Employed | | 6. Retired |
| **Current Living Arrangement** | 1.Joint Family | 2.Nuclear Family | | 3.Living Alone | 4. Living in Hostel PG/ Accommodation | | |
|  | 5.Living with Friends/ Family | | | 6.Homeless | | | |
| **Area of Residence** | 1.Rural | 2.Urban | | 3.Semiurban | 4.Slum Area | | |
| Education of Head of Family |  | Occupation of Head of Family | |  | Monthly Income of Family | |  |
| **Socio-Economic Class** | 1.Upper | 2.Upper Middle | | 3.Lower Middle | 4.Upper Lower | | 5.Lower |

| **Details of Past Medical/ Psychiatric History** | |
| --- | --- |
| Do you have any lifetime or current history of medical illness(es)? |  |
| Do you have any lifetime or current history of psychiatric illness(es)? |  |
| Do you have any lifetime or current history of neurological illness(es)? |  |
| Do you have any lifetime or current history of traumatic brain injury? |  |
| Do you have any lifetime or current history of any other illness(es)? |  |
| **Family History** | |
| Substance Use Disorder |  |
| Psychiatric Illness |  |
| Medical Illnesses |  |
| Neurological Illness |  |

| **Details of Substance Use** | | | | | | | | | | |
| --- | --- | --- | --- | --- | --- | --- | --- | --- | --- | --- |
| **Name of Substance** | **Category of Substance** | **Route of Use** | **Age of Onset of Use** | **Duration of Use** | **Usual Amount of Use** | **Usual Frequency of Use** | **Pattern of Use** | **No. of Abstinent Attempts**  **(AA)** | **Total Duration (AA)** | **Last Intake** |
|  |  |  |  |  |  |  |  |  |  |  |
|  |  |  |  |  |  |  |  |  |  |  |
|  |  |  |  |  |  |  |  |  |  |  |
|  |  |  |  |  |  |  |  |  |  |  |
|  |  |  |  |  |  |  |  |  |  |  |

| **Withdrawal Record** | |
| --- | --- |
| **Scale** |  |
| COWS |  |
| CWS |  |
| CIWA-Ar |  |
| CIWA-B |  |
| BRS |  |

| **Details of Gambling Behaviour** | | | | | | | | | | | |
| --- | --- | --- | --- | --- | --- | --- | --- | --- | --- | --- | --- |
| **Name of Gambling Behaviour** | **Category of Gambling Behaviour** | **Age of Onset of Gambling Behaviour** | **Duration of Gambling Behaviour** | **Usual Frequency of Gambling Behaviour**  **(Times)** | **Pattern of Gambling Behaviour** | **AA** | **Duration of AA** | **Last Engagement** | **Net Amount Spent** | **Net Loss** | **Net Profit** |
|  |  |  |  |  |  |  |  |  |  |  |  |
|  |  |  |  |  |  |  |  |  |  |  |  |
|  |  |  |  |  |  |  |  |  |  |  |  |
|  |  |  |  |  |  |  |  |  |  |  |  |
|  |  |  |  |  |  |  |  |  |  |  |  |

| **Treatment Record** | |
| --- | --- |
| **Medications** | **Dose/ Day (mg)** |
|  |  |
|  |  |
|  |  |
|  |  |
|  |  |
|  |  |
|  |  |
|  |  |
|  |  |
|  |  |
|  |  |
| **Other Treatments** | |
| **Treatment Name** | **Details** |
|  |  |
|  |  |
|  |  |
|  |  |
|  |  |
|  |  |
|  |  |
|  |  |
