# Supplementary material for: Protocol for a randomized controlled trial to evaluate the efficacy of inhibitory control training for aggressive behaviours among individuals with co-occurring substance use disorder and gambling behaviour
Source: Trials. 2026 Feb 6;27:199. doi: 10.1186/s13063-026-09503-y (PMC12973630; doi:10.1186/s13063-026-09503-y)
Supplement: Supplementary file 2 — Additional file 2. Details of the Primary and Secondary Outcome Measures. [file 13063_2026_9503_MOESM2_ESM.zip › Additional File 2R2.docx]

**Table S1:** Details of the Primary Outcome Measures

| **Outcome Measure** | **Measurement Variable** | **Analysis Metric** | **Method of Aggregation** | **Time Point for Each Outcome** |
| --- | --- | --- | --- | --- |
| Overt Aggression Scale – Modified (OAS-M)^[27,28]^ - Hindi Version | Overall Aggression Score (Frequency) | Change from baseline to 1-Month post intervention follow-up. | Mean (SD) | **Baseline**, **1 - Day post-intervention**, **1 - Month post-intervention (± 4 days),** and **3 - Months post-intervention (± 7 days).** |

**Table S2:** Details of the Secondary Outcome Measures

| **Outcome Measure** | **Measurement Variable** | **Analysis Metric** | **Method of Aggregation** | **Time Point for Each Outcome** |
| --- | --- | --- | --- | --- |
| Overt Aggression Scale – Modified (OAS-M)^[27,28]^ -Hindi Version | Verbal Aggression (Frequency)  Aggression Against Objects (Frequency)  Aggression Against Others (Frequency)  Aggression Against Self (Frequency)  Global Subjective Anger (Rating Score)  Global Overt Aggression (Rating Score)  Number of A1 Aggressive Episodes  Number of A2 Aggressive Episodes | Change from baseline to each follow-up. | Mean (SD)  Mean (SD)  Mean (SD)  Mean (SD)  Mean (SD)  Mean (SD)  Mean (SD)  Mean (SD) | **Baseline**, **1 - Day post-intervention**, **1 - Month post-intervention (± 4 days),** and **3 - Months post-intervention (± 7 days).** |
| Inhibitory Control Task | **Number of Commission Errors**  **Number of** Omission Errors | Change from baseline to each follow-up time point. | Mean (SD) | **Baseline**, **1 - Day post-intervention**, **1 - Month post-intervention (± 4 days),** and **3 - Months post-intervention (± 7 days).** |
| Stimulus Evaluation Task | G**eneralization Score** | Change from baseline to each follow-up time point. | Mean (SD) | **Baseline**, **1 - Day post-intervention**, **1 - Month post-intervention (± 4 days),** and **3 - Months post-intervention (± 7 days).** |
| Semi-Structured Performa for Details of Gambling Behaviour | Name(s) of Gambling Behaviour  Category of Gambling Behaviour  Age of Onset of Gambling Behaviour  Duration of Gambling Behaviour  Usual Frequency of Gambling Behaviour  Pattern of Gambling Behaviour  Total Number of Abstinent Attempts  Duration of Abstinent Attempts  Last Episode of Gambling Behaviour  Net Amount Spent in Gambling Behaviour  Net Loss in Gambling Behaviour  Net Profit in Gambling Behaviour | Change from baseline to each follow-up time point. | Proportion  Proportion  Mean (SD)  Mean (SD)  Mean (SD)  Proportion  Mean (SD)  Mean (SD)  Mean (SD)  Mean (SD)  Mean (SD)  Mean (SD) | **Baseline**, **1 - Month post-intervention (± 4 days),** and **3 - Months post-intervention (± 7 days).** |
| South Oaks Gambling Screen (SOGS)^[31]^ – Hindi Version | Severity of Gambling Behaviour  (Other Exploratory Variables to Consider)  Type of Gambling Activity  Mode of Gambling Activity  Frequency of Engagement  Social Consequences  Financial Consequences  Impact on Interpersonal Relationships  Attempts to Reduce or Stop Engagement | Change from baseline to each follow-up time point. | Mean (SD) | **Baseline**, **1 - Month post-intervention (± 4 days),** and **3 - Months post-intervention (± 7 days).** |
| Semi-Structured Performa for Details of Substance Use | Name(s) of Substance  Category of Substance  Age of Onset of Use  Duration of Use  Usual Amount of Use  Usual Frequency of Use  Pattern of Use  Number and Duration of Abstinent Attempts  Last Intake | Change from baseline to each follow-up time point. | Proportion  Proportion  Mean (SD)  Mean (SD)  Mean (SD)  Mean (SD)  Proportion  Mean (SD)  Mean (SD) | **Baseline**, **1 - Month post-intervention (± 4 days),** and **3 - Months post-intervention (± 7 days).** |
| Timeline Follow-Back Scale (TLFB)^[32]^ | Retrospective recall of quantity and frequency of substance intake over the last month. | Change from baseline to each follow-up time point. | Mean (SD) | **Baseline**, **1 - Month post-intervention (± 4 days),** and **3 - Months post-intervention (± 7 days).** |
| Alcohol, Smoking and Substance Involvement Screening Test (ASSIST V3.0) | Substance Involvement Score (Tobacco, Alcohol, Cannabis, Cocaine, Amphetamines, Inhalants, Sedatives, Hallucinogens, Opioids, Others) | Change from baseline to each follow-up time point. | Mean (SD) | **Baseline**, **1 - Month post-intervention (± 4 days),** and **3 - Months post-intervention (± 7 days).** |

*The final approach to the analysis and reporting of these variables, including exploration of changes from baseline to each follow-up time point, will be determined after confirming that the characteristics of the collected data are appropriate for the planned analyses.*
