# Supplementary material for: Protocol for a randomized controlled trial to evaluate the efficacy of inhibitory control training for aggressive behaviours among individuals with co-occurring substance use disorder and gambling behaviour
Source: Trials. 2026 Feb 6;27:199. doi: 10.1186/s13063-026-09503-y (PMC12973630; doi:10.1186/s13063-026-09503-y)
Supplement: Supplementary file 4 — Additional file 4. Informed consent documents given to the participants. [file 13063_2026_9503_MOESM4_ESM.docx]

## **Participant Information Sheet**

**Study Title:** A Randomized Controlled Trial to Evaluate the Efficacy of Inhibitory Control Training for Aggressive Behaviours among Individuals with Co-occurring Substance Use Disorder and Gambling Behaviour

**Investigators**

Ms. Yashita Ahluwalia (Phone No.: 01126593236)

Dr. Yatan Pal Singh Balhara (Phone No.: 01126593236)

**Participation in the Study:** You're invited to participate voluntarily in a research study focused on providing a psychological intervention aimed at alleviating symptoms induced by substance use through visual pictures. It is important for you to understand the purpose of this study and its procedures before deciding to participate. Please carefully review the following information. If anything is unclear to you or if you require additional details, please don't hesitate to ask. If you choose to participate, you will be asked to sign and date a consent form.

**Purpose**: This study aims to assess the effectiveness of a psychological intervention on reducing symptoms induced by substance use.

**Study Procedure:** During this study, you will undergo a 3-day intervention training or a sham training programme consisting of six 15-minute sessions, with two sessions being held on each day. You will not receive information in advance regarding whether you will undergo the sham or the intervention training. Before and after the training, we will assess your substance use, gambling behavior, and aggressive behaviors. Additionally, your performance on a task similar to the training will be evaluated. Follow-up assessments will occur at 1-month and 3-month intervals to test both the induced and sustained effects of the intervention. You will be required to provide a reachable contact number for communication purposes for the same.

**Expected duration of participation:** Your participation in the intervention will span 90 minutes, with sessions lasting up to 30 minutes each day over a period of 3 days. The pre-and post-assessments, as well as follow-up assessments, are anticipated to require approximately 50 minutes each.

**Benefits to be expected from the research to the subject or others:** While you will not directly benefit from participating in this study, the data gathered will contribute to advancing our scientific understanding on the efficacy and application of this psychological intervention, benefiting individuals with similar issues in the future.

**Any risk to the subject associated with the study:** There is no risk associated with the study or any presumptive loss identified due to participation in the study.

**Maintenance of Confidentiality of Records:** Your identity and all data collected from you will be kept strictly confidential. Your information will not be disclosed to any party outside of the study, nor will it be used for any purpose other than publication related to the study. We will maintain a password-protected computer system to ensure the security of your data. Anonymity and confidentiality will be rigorously upheld throughout the study and during the publication process.

**Provision of free treatment for research-related injury:** There is no such significantly increased of injury as a result of participation in the study.

**Compensation of subject for disability or death resulting from such injury:** Participation in this study will not incur any costs, and you will not receive any financial compensation for your involvement. Additionally, there are no provisions for free treatment in the event of any research-related injury, although the risk of such injury is not significantly increased by participation in the study.

**Freedom of individual to participate and to withdraw from study at any time without penalty or loss of benefits to which the subject would otherwise be entitled**: You will have the right to refuse participation in the study or withdraw at any time, and this decision will not impact your treatment at NDDTC, AIIMS. Your well-being and treatment remain our priority, regardless of your involvement in the study.

**Amount of blood sample in quantity to be taken should be mentioned:**

NA

**Costs and source of investigations, disposables, implants and drugs/ contrast media should be mentioned:**

NA

**Telephone/ Contact number of Principle Investigator and Co-Investigator at the top of each page:**

You are free to contact the investigators for clarifications.

**Statement that there is a possibility of failure of IP to provide intended therapeutic effect:**

Please be advised that although the psychological intervention is designed to deliver therapeutic benefits, it is important to acknowledge the possibility of it not achieving the intended effects. While the investigational product aims to deliver therapeutic benefits, there exists the possibility, as with any other intervention, that it may not achieve the intended effects for every participant.

**Statement that in case if placebo-controlled trails, the placebo administered to the subjects shall not gave any therapeutic effect**: Our placebo is a sham-controlled training, meaning it is designed to mimic the active intervention without providing any therapeutic benefit.

- 1. **Date: Investigator’s Signature:**

**प्रतिभागी सूचना पत्रक**

**अध्ययन का शीर्षक:** अध्ययन का शीर्षक: सह-पदार्थ उपयोग विकार और जुआ व्यवहार वाले व्यक्तियों के बीच आक्रामक व्यवहार के लिए निरोधात्मक नियंत्रण प्रशिक्षण की प्रभावशीलता का मूल्यांकन करने के लिए एक यादृच्छिक नियंत्रित परीक्षण

**अन्वेषक**

सुश्री यशिता अहलूवालिया (फोन नंबर: 01126593236)

डॉ. यतन पाल सिंह बल्हारा (फोन नंबर: 01126593236)

**अध्ययन में भागीदारी:** आपको दृश्य चित्रों के माध्यम से नशे के उपयोग से प्रेरित लक्षणों को कम करने के उद्देश्य से मनोवैज्ञानिक इलाज प्रदान करने पर केंद्रित एक शोध अध्ययन में स्वेच्छा से भाग लेने के लिए आमंत्रित किया जाता है। भाग लेने का निर्णय लेने से पहले आपके लिए इस अध्ययन के उद्देश्य और इसकी प्रक्रियाओं को समझना महत्वपूर्ण है। कृपया निम्नलिखित जानकारी की सावधानीपूर्वक समीक्षा करें। यदि आपके लिए कुछ भी अस्पष्ट है या यदि आपको अतिरिक्त विवरण की आवश्यकता है, तो कृपया पूछने में संकोच न करें। यदि आप भाग लेना चुनते हैं, तो आपसे एक सहमति प्रपत्र पर हस्ताक्षर करने और तारीख बताने के लिए कहा जाएगा।

**उद्देश्य:** इस अध्ययन का उद्देश्य मादक द्रव्यों के उपयोग से प्रेरित लक्षणों को कम करने पर मनोवैज्ञानिक हस्तक्षेप की प्रभावशीलता का आकलन करना है।

**अध्ययन प्रक्रिया:** इस अध्ययन के दौरान, आपको 3-दिवसीय हस्तक्षेप प्रशिक्षण या एक दिखावटी प्रशिक्षण कार्यक्रम से गुजरना होगा जिसमें छह 15-मिनट के सत्र होंगे, जिसमें प्रत्येक दिन दो सत्र होंगे। आपको इस संबंध में पहले से जानकारी नहीं मिलेगी कि आप दिखावा या हस्तक्षेप प्रशिक्षण से गुजरेंगे या नहीं। प्रशिक्षण से पहले और बाद में, हम आपके मादक द्रव्यों के उपयोग, जुआ व्यवहार और आक्रामक व्यवहार का आकलन करेंगे। इसके अतिरिक्त, प्रशिक्षण के समान कार्य पर आपके प्रदर्शन का मूल्यांकन किया जाएगा। हस्तक्षेप के प्रेरित और निरंतर दोनों प्रभावों का परीक्षण करने के लिए अनुवर्ती मूल्यांकन 1 महीने और 3 महीने के अंतराल पर होंगे। आपको इसके लिए संचार उद्देश्यों के लिए एक पहुंच योग्य संपर्क नंबर प्रदान करना आवश्यक होगा।

**भागीदारी की अपेक्षित अवधि:** उपचार में आपकी भागीदारी 90 मिनट तक चलेगी, जिसमें 3 दिनों की अवधि में प्रत्येक दिन 30 मिनट तक सत्र चलेंगे। पूर्व और बाद के मूल्यांकन, साथ ही अनुवर्ती मूल्यांकन, प्रत्येक में लगभग 50 मिनट की आवश्यकता होने का अनुमान है।

**शोध से विषय या अन्य लोगों को अपेक्षित लाभ:** हालांकि आपको इस अध्ययन में भाग लेने से सीधे लाभ नहीं होगा, लेकिन एकत्र किया गया डेटा इस मनोवैज्ञानिक उपचार की प्रभावकारिता और अनुप्रयोग के बारे में हमारी समझ को आगे बढ़ाने में योगदान देगा, जिससे समान मुद्दों वाले व्यक्तियों को लाभ होगा।

**अध्ययन से जुड़े विषय के लिए कोई जोखिम:** अध्ययन से जुड़ा कोई जोखिम नहीं है या अध्ययन में भाग लेने के कारण कोई अनुमानित हानि की पहचान नहीं की गई है।

**रिकॉर्ड्स की गोपनीयता बनाए रखना:** आपकी पहचान और आपसे एकत्र किए गए सभी डेटा को सख्ती से गोपनीय रखा जाएगा। आपकी जानकारी अध्ययन के बाहर किसी भी पक्ष को प्रकट नहीं की जाएगी, न ही इसका उपयोग अध्ययन से संबंधित प्रकाशन के अलावा किसी अन्य उद्देश्य के लिए किया जाएगा। हम आपके डेटा की सुरक्षा सुनिश्चित करने के लिए एक पासवर्ड-सुरक्षित कंप्यूटर सिस्टम बनाए रखेंगे। पूरे अध्ययन के दौरान और प्रकाशन प्रक्रिया के दौरान गुमनामी और गोपनीयता को सख्ती से बरकरार रखा जाएगा।

**शोध-संबंधी चोट के लिए निःशुल्क उपचार का प्रावधान:** अध्ययन में भागीदारी के परिणामस्वरूप चोट में कोई उल्लेखनीय वृद्धि नहीं हुई है।

**ऐसी चोट के परिणामस्वरूप विकलांगता या मृत्यु के लिए विषय का मुआवजा:** इस अध्ययन में भाग लेने पर कोई लागत नहीं आएगी, और आपको अपनी भागीदारी के लिए कोई वित्तीय मुआवजा नहीं मिलेगा। इसके अतिरिक्त, किसी भी शोध-संबंधी चोट की स्थिति में मुफ्त उपचार का कोई प्रावधान नहीं है, हालांकि अध्ययन में भाग लेने से ऐसी चोट का जोखिम बहुत अधिक नहीं बढ़ता है।

**व्यक्ति को किसी भी समय अध्ययन में भाग लेने और बिना दंड या लाभ की हानि के, जिसका विषय अन्यथा हकदार होगा, अध्ययन से हटने की स्वतंत्रता:** आपके पास किसी भी समय अध्ययन में भाग लेने से इनकार करने या वापस लेने का अधिकार होगा, और यह निर्णय नहीं होगा एनडीडीटीसी, एम्स में आपके इलाज पर असर। अध्ययन में आपकी भागीदारी चाहे जो भी हो, आपकी भलाई और उपचार हमारी प्राथमिकता बनी हुई है।

**लिए जाने वाले रक्त के नमूने की मात्रा का उल्लेख किया जाना चाहिए:**

ना

**जांच, डिस्पोजल, इम्प्लांट और दवाओं/कंट्रास्ट मीडिया की लागत और स्रोत का उल्लेख किया जाना चाहिए:**

ना

**प्रत्येक पृष्ठ के शीर्ष पर प्रमुख अन्वेषक और सह-अन्वेषक का टेलीफोन/संपर्क नंबर:**

आप स्पष्टीकरण के लिए जांचकर्ताओं से संपर्क करने के लिए स्वतंत्र हैं।

**यह कथन कि इच्छित चिकित्सीय प्रभाव प्रदान करने में आईपी की विफलता की संभावना है:**

कृपया ध्यान रखें कि यद्यपि मनोवैज्ञानिक हस्तक्षेप चिकित्सीय लाभ प्रदान करने के लिए डिज़ाइन किया गया है, लेकिन इसके इच्छित प्रभावों को प्राप्त नहीं करने की संभावना को स्वीकार करना महत्वपूर्ण है। जबकि जांच उत्पाद का लक्ष्य चिकित्सीय लाभ पहुंचाना है, किसी भी अन्य हस्तक्षेप की तरह, यह संभावना मौजूद है कि यह प्रत्येक भागीदार के लिए इच्छित प्रभाव प्राप्त नहीं कर सकता है।

**कथन कि यदि प्लेसबो-नियंत्रित ट्रेल्स हैं, तो विषयों को प्रशासित प्लेसबो कोई चिकित्सीय प्रभाव नहीं देगा:** हमारा प्लेसबो एक दिखावटी-नियंत्रित प्रशिक्षण है, जिसका अर्थ है कि इसे कोई चिकित्सीय लाभ प्रदान किए बिना सक्रिय हस्तक्षेप की नकल करने के लिए डिज़ाइन किया गया है।

**तारीख: अन्वेषक के हस्ताक्षर:**

**Participant Informed Consent Form**

Protocol No.: ....................... Participant Identification Number: .....................

**Study Title:** A Randomized Controlled Trial to Evaluate the Efficacy of Inhibitory Control Training for Aggressive Behaviours among Individuals with Co-occurring Substance Use Disorder and Gambling Behaviour

**Investigators**

Ms. Yashita Ahluwalia (Phone No. – 01126593236)

Dr. Yatan Pal Singh Balhara (Phone No. – 01126593236)

The contents of the information sheet dated ........................... that was provided have been read carefully by me / explained in detail to me, in a language that I comprehend, and I have fully understood the contents. I confirm that I have had the opportunity to ask questions. The nature and purpose of the study and its potential risks/benefits and expected duration of the study and other relevant details of the study have been explained to me in detail. I understand that my participation is voluntary and that I am free to withdraw at any time, without giving any reason, without my medical care or legal right being affected.

I understand that the information collected about me from my participation in this research and sections of any of my medical notes may be looked at by responsible individuals from AIIMS. I give permission for these individuals to have access to my records. I give my full, free and willing consent to take part in the above study.

_____________________________ Date:

(Signature / Left Thumb Impression) Place:

Name of the Participant:

Son / Daughter / Spouse of:

Complete Postal Address:

This is to certify that the above consent has been obtained in my presence.

_______________________ Date:

(Signature of the Investigator) Place:

1) Witness – 1 2) Witness – 2

(Signature) (Signature)

Name: ..................................... Name: .....................................

Address: ................................. Address: .................................

## **प्रतिभागी सूचित सहमति पत्र**

प्रोटोकॉल संख्या: ....................... प्रतिभागी पहचान संख्या: ....................

**अध्ययन का शीर्षक:** अध्ययन का शीर्षक: सह-पदार्थ उपयोग विकार और जुआ व्यवहार वाले व्यक्तियों के बीच आक्रामक व्यवहार के लिए निरोधात्मक नियंत्रण प्रशिक्षण की प्रभावशीलता का मूल्यांकन करने के लिए एक यादृच्छिक नियंत्रित परीक्षण

**अन्वेषक**

सुश्री यशिता अहलूवालिया (फोन नंबर: 01126593236)

डॉ. यतन पाल सिंह बल्हारा (फोन नंबर: 01126593236)

इस सूचना पत्र की सामग्री जो दिनांक . . . . . . . को प्रदान की गई थी, मैंने सावधानीपूर्वक पढ़ लिया है/मुझे उस भाषा में विस्तार से समझा दिया गया है जो मुझे समझ में आती है और मैंने पूरी सामग्री को अच्छी तरह समझ लिया है। मैं पुष्टि करता हूं कि मुझे प्रश्न पूछने का अवसर दिया गया है। अध्ययन का प्रकार और प्रयोजन तथा इसके संभावित जोखिम/लाभ और अध्ययन पूरा होने की अनुमानित अवधि तथा अध्ययन के अन्य संगत विवरण मुझे विस्तार से समझा दिया गए हैं। मुझे बताया गया है कि मेरी भागीदारी स्वेच्छानुसार है और मैं कोई कारण बताए बिना किसी भी समय वापस जाने के लिए पूरी तरह स्वतंत्र हूं और इस पर मेरी चिकित्सा देखभाल या कानूनी अधिकारों पर कोई प्रभाव नहीं पड़ेगा।

मुझे पता है कि इस अनुसंधान में मेरी भागीदारी के बारे में जमा की गई जानकारी एम्स के जिम्मेदार व्यक्तियों द्वारा देखी जाएगी, जहां इस अनुसंधान में मेरे भाग लेने को संगत पाया जाए। मैं इन व्यक्तियों को अपने अभिलेख देखने की अनुमति देता हूं। मैं उपरोक्त अध्ययन में भाग लेने के लिए अपनी पूर्ण**,** स्वतंत्र और इच्छुक सहमति देता हूं

_______________________ दिनांक:

(हस्ताक्षर/बाएं अंगूठे का निशान) स्थान:

प्रतिभागी का नाम:

पुत्र/पुत्री/जीवन साथी:

डाक का पूरा पता :

________________________ दिनांक:

(हस्ताक्षर/बाएं अंगूठे का निशान) स्थान:

1) गवाह - 1 2) गवाह - 2

हस्ताक्षर; हस्ताक्षर:

नाम: नाम :

पता: पता:
